# Deep neural networks for A-line-based plaque classification in coronary intravascular optical coherence tomography images

**DOI:** 10.1117/1.JMI.5.4.044504

**Published:** 2018-12-03

**Authors:** Chaitanya Kolluru, David Prabhu, Yazan Gharaibeh, Hiram Bezerra, Giulio Guagliumi, David Wilson

**Affiliations:** a Case Western Reserve University, Department of Biomedical Engineering, Cleveland, Ohio, United States; b University Hospitals, Harrington Heart and Vascular Institute, Cardiovascular Imaging Core Laboratory, Cleveland, Ohio, United States; c Ospedali Riuniti di Bergamo, Cardiovascular Department, Bergamo, Italy; d Case Western Reserve University, Department of Radiology, Cleveland, Ohio, United States

**Keywords:** optical coherence tomography, pattern recognition, neural networks

## Abstract

We develop neural-network-based methods for classifying plaque types in clinical intravascular optical coherence tomography (IVOCT) images of coronary arteries. A single IVOCT pullback can consist of >500 microscopic-resolution images, creating both a challenge for physician interpretation during an interventional procedure and an opportunity for automated analysis. In the proposed method, we classify each A-line, a datum element that better captures physics and pathophysiology than a voxel, as a fibrous layer followed by calcification (fibrocalcific), a fibrous layer followed by a lipidous deposit (fibrolipidic), or other. For A-line classification, the usefulness of a convolutional neural network (CNN) is compared with that of a fully connected artificial neural network (ANN). A total of 4469 image frames across 48 pullbacks that are manually labeled using consensus labeling from two experts are used for training, evaluation, and testing. A 10-fold cross-validation using held-out pullbacks is applied to assess classifier performance. Noisy A-line classifications are cleaned by applying a conditional random field (CRF) and morphological processing to pullbacks in the *en-face* view. With CNN (ANN) approaches, we achieve an accuracy of 77.7%±4.1% (79.4%±2.9%) for fibrocalcific, 86.5%±2.3% (83.4%±2.6%) for fibrolipidic, and 85.3%±2.5% (82.4%±2.2%) for other, across all folds following CRF noise cleaning. The results without CRF cleaning are typically reduced by 10% to 15%. The enhanced performance of the CNN was likely due to spatial invariance of the convolution operation over the input A-line. The predicted *en-face* classification maps of entire pullbacks agree favorably to the annotated counterparts. In some instances, small error regions are actually hard to call when re-examined by human experts. Even in worst-case pullbacks, it can be argued that the results will not negatively impact usage by physicians, as there is a preponderance of correct calls.

## 1 Introduction

Cardiovascular disease is the leading cause of death globally, accounting for more than 15% of all deaths in 2015. Coronary atherosclerosis is the process of plaque buildup in the coronary arteries. To relieve narrowing of an obstructed coronary artery, physicians often perform percutaneous coronary interventions (PCIs), which involve revascularization procedures, such as balloon angioplasty and stent treatment. Although x-ray angiography is commonly used to guide such interventions, this imaging technique can only indicate luminal narrowing due to the presence of calcium deposits but does not render any further information about the vessel wall. Nonetheless, intravascular imaging techniques can aid cardiologists in treatment planning for the majority of PCI cases. To aid the physician in such a scenario, we developed a coronary plaque classification system based on intravascular optical coherence tomography (IVOCT) images.

IVOCT is a high-contrast, high-resolution, imaging technique that can be used to characterize various atherosclerotic plaque types and guide stent placement.^1^ As compared with intravascular ultrasound, IVOCT has higher resolution, improved imaging through a calcification, and better visual discrimination between fibrous and lipid tissues.^2^ In addition, IVOCT is currently the only imaging technique that allows identification of vulnerable thin-cap fibroatheromas, which have been identified as the most susceptible to rupture.^3^ IVOCT is also useful for the planning of stent interventions in the presence of significant calcium or lipid deposits.^4^ Despite these obvious advantages of IVOCT for treatment planning, physician enthusiasm has been tempered by the need for specialized training to interpret IVOCT images and the overload of image data generated from a single pullback, which often results in >500 images from a single 2- to 5-s scan.

Buoyed by the success and interest in human expert evaluation, research on the machine identification of plaque types has achieved considerable success with the development of both voxel- and A-line-based classification techniques. For example, Ughi et al.^5^ developed a voxel-based classification scheme that combined geometric and textural features along with a sliding window approach to estimate the attenuation coefficient value of each voxel directly from IVOCT images. This method has achieved an overall classification accuracy of 81.5%. Athanasiou et al.^6^ used a K-means algorithm to obtain an initial clustering of pixels within an image and then extracted various textural and intensity features to classify individual clusters into one of four plaque types, namely, fibrous tissue, lipid tissue, calcium, and mixed tissue. They report a computation time of 40 s per image frame, prohibiting real-time use. Rico-Jimenez et al.^7^ devised an A-line-based classification technique by modeling an A-line as a linear combination of a number of depth profiles with the use of morphological features to perform the classification. This approach is useful for plaque classification because physicians are primarily interested in determining the location and length of the stent for a particular case. We believe that the A-line-based classification technique provides sufficient information for the clinician to make both of these decisions simultaneously with ease. However, the method described in this prior report was applied only to fibrotic and lipid plaques without consideration of calcium plaques. In addition to fully automated methods, such as those described already, semiautomated approaches for calcified plaque segmentation have also been developed. For example, Wang et al.^8^ described a semiautomatic algorithm that requires user input of start and stop frames within the pullback. Again, A-line-based classification would allow seamless integration into such semiautomatic segmentation methods.

A recent survey conducted by Boi et al.^9^ described the large potential of leveraging deep learning techniques for atherosclerotic plaque characterization and subsequent risk stratification using IVOCT. The use of deep neural networks was recently reported by Abdolmanafi et al.^10^ for the identification of layers within the coronary artery wall. Although this was the first study to use deep learning in the context of tissue characterization in IVOCT, the analysis was limited to the detection of the coronary artery layers without plaque classification.

In this study, a learning system [convolutional neural network (CNN) and fully connected artificial neural network (ANN)] was applied for the classification of coronary plaques. Rather than using voxel-based classification, A-lines were used as the fundamental unit because the many attributes within an A-line (e.g., sharp transitions at the edge of a calcification and the large signal decay with depth in a lipid region) will likely contribute to the classification of coronary plaques. Because clinical interest is expected to extend to larger regions and classification can be noisy, a fully connected conditional random field (CRF) method^11^ was employed to standardize classification over larger regions. The proposed system was trained and tested on a carefully labeled dataset from 48 IVOCT pullbacks, containing nearly 4500 images and more than two million A-lines.

## 2 Image Analysis Methods

Image processing and learning techniques, either an ANN or deep CNN, were applied to classify A-lines in IVOCT images as fibrocalcific, fibrolipidic, or other. The naming convention that we will use for the rest of this paper is as follows: fibrocalcific A-line refers to an A-line with a fibrous layer followed by calcification, and fibrolipidic A-line refers to a fibrous layer followed by a lipidous deposit. The algorithm can be broken down into three main steps: (1) preprocessing, which includes lumen boundary detection, alignment of tissues via pixel shifting, and noise reduction; (2) deep neural network for classification of individual A-lines; and (3) classification noise cleaning using a CRF and morphological processing in the *en-face* (θ,z) classification view.

### 2.1 Preprocessing

Preprocessing steps were applied to raw IVOCT images obtained in the polar (r,θ) domain. First, the lumen boundary was located on the image using a dynamic programming approach previously developed by our group.^8^ Briefly, the edges along the radial direction were identified by filtering the image, and then, dynamic programming was used to identify the contour with the greatest cumulative edge strength along the angular direction θ, as the lumen boundary. Second, the position of the guidewire shadow was located using a previously described method,^12^ and the A-line values within the guidewire region were set to zero. Third, A-lines were pixel shifted along the radial direction so that the first pixel in each row corresponded to the first pixel after the lumen boundary on the original image. This step was added to properly align tissues in images acquired with an eccentrically located catheter. Fourth, only the first 200 pixels (∼1  mm) of the vessel wall for each A-line were used, and the others were cropped, because IVOCT has limited penetration into tissue. Fifth, a log transformation was applied to convert multiplicative speckle into an additive form. Sixth, speckle was reduced by filtering with a Gaussian kernel with a size of (7, 7) and standard deviation of 1. The baseline subtraction and roll-off correction step as described in Ref. 13 was not performed because the goal of this method was to classify A-lines rather than estimate the attenuation coefficients of tissues.

### 2.2 Convolutional Neural Network and Artificial Neural Network Systems

**Fig. 1:**
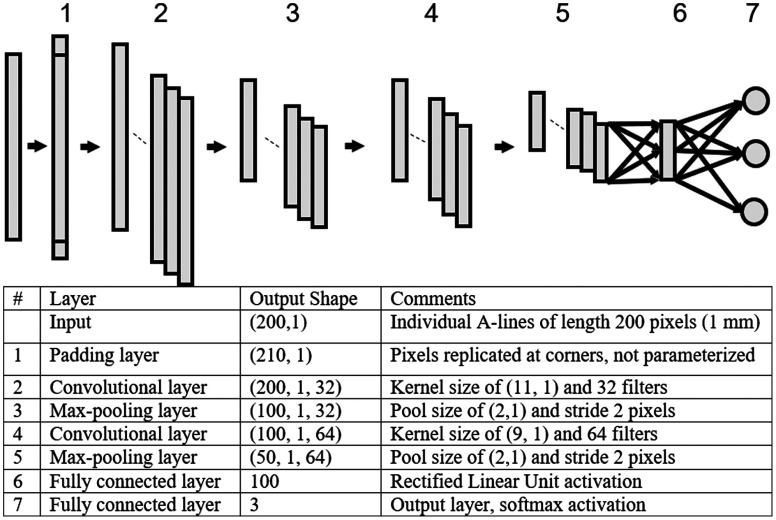
CNN architecture. The network consists of seven layers excluding the input layer. The details regarding the individual layers in this network are described in the table within the figure and in text.

The deep CNN used individual A-lines as input and output probability values for each class. The architecture contained seven layers, as shown in Fig. 1. Layer 1 padded the front and back edges of the input A-line (along r) by 5 pixels by replicating the first and last pixel. Such padding allows for the subsequent convolution operation to generate a feature map of the same size as the input A-line, which is important because the fibrous caps of a vulnerable plaque can be thin (<65  μm or 13 pixels). In layer 2, a convolutional layer was employed that learns 32 filters, each with a length of 11 pixels. Layer 3 performs a maximum pooling operation with a pool size of 2 pixels and stride of 2 pixels. Layer 4 uses another convolutional layer that learns 64 filters, each with a length of 9 pixels. Layer 5 consisted of another maximum pooling operation with a pool size of 2 pixels and stride of 2 pixels. Layer 6 consists of a fully connected network of 100 units with the rectified linear unit as the nonlinear activation function. Layer 7 is a fully connected layer of three units with a softmax activation function to generate probability values for each class that sum to 1.

In addition, a fully connected ANN was applied, and its performance was compared with that of the CNN. The ANN takes each individual pixel in an A-line as input. Two hidden layers of 100 and 50 units were connected to a fully connected output layer of three units corresponding to the three classes of interest. The rectified linear unit function is used as the activation function for both hidden layers. The final output layer uses softmax activation. The architecture of the ANN is described in Table 1. As described in Sec. 3.2, some modifications were made to these basic networks and standard methods were utilized to aid in training.

**Table 1:** ANN architecture.

No.	Layer	Output shape	Comments
	Input	(200, 1)	Individual A-lines of length 200 pixels (1 mm)
1	Hidden layer	100	Fully connected, rectified linear unit activation
2	Hidden layer	50	Fully connected, rectified linear unit activation
3	Output layer	3	Output layer, softmax activation

To better understand the most important features for classification, saliency maps were created using the guided backpropagation method described by Springenberg et al.^14^ to identify the pixels in an A-line that were most responsible for the output for a specific class. The reconstructed saliency map is thus both class and image specific. Briefly, the method computes a forward pass of the image (A-line in this case) through the trained classification network and then performs a backward pass, that is, computes the gradient of the class activation with respect to the input image. A large magnitude of the gradient indicates that a small change to such pixels would have a large impact on the class activation value and, therefore, the network prediction. Maps were created for individual A-lines and grouped across all A-lines in an image to create a visualization over a full two-dimensional (2-D) IVOCT image.

### 2.3 Classification Noise Cleaning

Because individual A-line classification results are noisy when viewed across a pullback, cleaning of classification noise was employed as a postprocessing step. A method to integrate network outputs to a fully connected CRF is described in Ref. 15. Here, a set of classified A-lines across consecutive frames within an IVOCT pullback is defined as a lesion segment. For each lesion segment, an *en-face* 2-D “image” of classification results in (θ,z) was constructed where each pixel contains the vector of class probabilities for the corresponding A-line. The task of the CRF is to reduce noise by generating a new labeling that favors assigning the same label to pixels that are closer to each other spatially (both in θ and z) using the probability estimates generated by the neural network.

A CRF is an undirected graphical model that encodes a conditional distribution over the target variable Y given a set of the observed variable X. This method maximizes the distribution P(Y|X), which is expressed as a Gibbs distribution over a random field. The fully connected CRF described in Ref. 11 computes the maximum a posteriori label by minimizing the energy function as follows:

$$E(l)=\sum_{i}\theta_{i}(l_{i})+\sum_{i}\theta_{i,j}(l_{i},l_{j}),$$

where l is a particular label assignment for all pixels in the image; $\theta_{i}(l_{i})=-\log\;P(l_{i})$ is the unary potential, where $P(l_{i})$ is the probability estimate of label l at pixel i computed by the neural network; $\theta_{i,j}(l_{i},\;l_{j})$ is the pairwise edge potential that connects all pixel pairs in the image i, j and is defined as a linear combination of Gaussian kernels as shown as follows:

$$\theta_{i,j}(l_{i},l_{j})=\mu (l_{i},l_{j})[w_{1}\;\exp(-\frac{\left\|p_{i}-p_{j}^{2}\right\|}{2\sigma_{\alpha }^{2}}-\frac{\left\|I_{i}-I_{j}^{2}\right\|}{2\sigma_{\beta }^{2}})+w_{2}\;\exp(-\frac{\left\|p_{i}-p_{j}^{2}\right\|}{2\sigma_{\gamma }^{2}})],$$

where the label compatibility function $\mu (l_{i},l_{j})=1$ if $l_{i}\neq l_{j}$ and zero otherwise; $p_{i}$ and $p_{j}$ refer to the spatial positions of pixels i and j, respectively; $I_{i}$ and $I_{j}$ indicate the intensity vectors of pixels i and j, respectively; $w_{1}$ and $w_{2}$ are the weights of the appearance and smoothness terms, respectively; and $\sigma_{\alpha }$, $\sigma_{\beta }$, and $\sigma_{\gamma }$ control the degree of interaction either in the spatial or intensity dimensions. Because pixels in the *en-face* image do not have a specific intensity value, this pairwise potential was modified by dropping the appearance kernel term ($w_{1}$ is set to zero), which then leaves the smoothness kernel, yielding the following pairwise potential term:

$$\theta_{i,j}(l_{i},l_{j})=\mu (l_{i},l_{j})[w_{2}\;\exp(-\frac{\left\|p_{i}-p_{j}^{2}\right\|}{2\sigma_{\gamma }^{2}})],$$

where $\left\|p_{i}-p_{j}\right\|$ is the spatial distance between pixels i and j, and $\sigma_{\gamma }$ controls the size of the smoothness kernel. A mean field approximation was used for inference that minimizes the Kullback-Leibler-divergence between P(Y|X) and a fully factorable distribution Q. The message passing step within the iterative update scheme can be expressed as a Gaussian filtering rendering the algorithm computationally efficient. Three free parameters are left with for the CRF: the size of the smoothness kernel, $\sigma_{\gamma }$, weight of the smoothness kernel $w_{2}$, and the number of iterations, n. Overall, for each pixel in the *en-face* A-line classification view, the CRF takes in probability estimates of each class as input and outputs its final class ownership.

Finally, three iterations of an area opening operation with an area threshold of 10 pixels are performed serially on the *en-face* view images. Each iteration considers one of the three classes as the background class and the remaining two as the foreground class. This step closes small holes within fibrocalcific and fibrolipidic chunks and removes small islands containing these plaques.

## 3 Experimental Methods

### 3.1 Labeled Image Data

A dataset of clinical *in vivo* IVOCT pullbacks from 48 patients was obtained from the TRANSFORM clinical trial.^16^ All pullbacks were imaged prior to the interventional procedure. IVOCT images were collected using a frequency-domain OCT system (Ilumien Optis; St. Jude Medical, St. Paul, Minnesota), which consists of a tunable laser light source sweeping from 1250 to 1360 nm at a frame rate of 180 fps, a pullback speed of 36 mm/s, and an axial resolution of about 20  μm. The pullbacks were analyzed by two expert readers in the Cartesian (x,y) view. In all, a total of 4469 image frames were analyzed across 48 pullbacks. Labels from (x,y) images were converted back to the polar (r,θ) system for processing. Each polar (r,θ) image consisted of either 448 or 496 A-lines, 968 pixels along each A-line, and 16 bits of gray scale data per pixel.

**Fig. 2:**
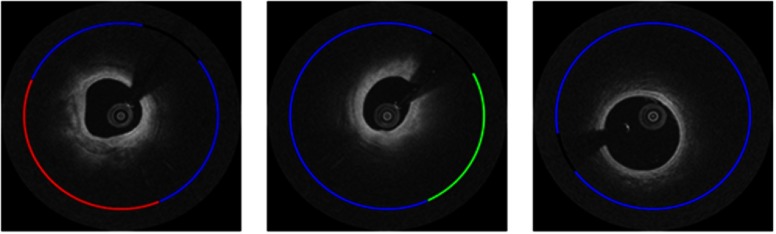
Example IVOCT images with A-line labels. The color code is red (fibrocalcific), green (fibrolipidic), blue (other), and black (guidewire shadow). Labels were created by consensus between two expert IVOCT readers.

Ground truth annotations were obtained by consensus of two expert IVOCT readers who were trained in the Cardiovascular Imaging Core Lab of the Harrington Heart and Vascular Institute (Cleveland, Ohio), a laboratory that has conducted numerous studies requiring expert reading of IVOCT images. The definitions in the consensus document were used to determine the ground truth labels, as described in Ref. 1. For example, a fibrocalcific plaque appears as a high backscattering and relatively homogeneous region (fibrous tissue) followed by a signal-poor region with sharply delineated front and/or back borders (calcium) on IVOCT images. A fibrolipidic region was defined as fibrous tissue followed by fast signal drop-off with diffuse borders corresponding to the presence of a necrotic core or lipid pool. The additional class “other” was used to include all A-lines that did not meet the criteria of the former two categories. Some example annotations are shown in Fig. 2.

### 3.2 Network Training and Testing

A 10-fold cross-validation procedure was used to measure classifier performance. Of 48 annotated pullbacks, 38 were randomly selected for training, 5 for validation, and 5 for testing in each iteration. The last iteration consisted of 40 pullbacks for training, 5 for validation, and 3 for testing. In this manner, each pullback was assigned into the test (leave-out) set exactly once. Mean and standard error of classification accuracy over the 10 iterations are reported.

We used the categorical cross-entropy function as the loss function that was minimized during network training. For a given example, categorical cross entropy was evaluated as L=−∑i∈Cyi log(y^i), where y is a one hot vector representation of the ground truth labels and y^ is the vector of probabilities computed by the neural network over C different classes. A class weighting scheme was employed during network training to account for class imbalance. Class weights were computed as the inverse of the class proportions in the training set and were used to weight the loss function. The weights are usually around 4, 4, and 1 for the fibrocalcific, fibrolipidic, and other classes, respectively. Network optimization was performed using the Adam optimizer^17^ with a learning rate of $1\times 10^{-4}$. In addition, because deep networks tend to overfit when trained for a large number of epochs, a validation set was used. Training was stopped when the loss of the validation dataset did not improve by more than 0.01% for 5 consecutive epochs or when the network was trained for 100 epochs, whichever occurred first. The model with the least validation loss during training was used to make predictions on the test set.

Preprocessing steps were performed using MATLAB^®^ R2016a software (MathWorks, Natick, Massachusetts). The Keras functional application programming interface with the TensorFlow machine learning framework as backend was used to implement, train, and test the neural networks with the given dataset. Network training was performed using two NVIDIA Tesla P100 graphics processing unit (GPU) cards.

The neural network architectures described in Sec. 2.2, namely, ${\mathrm{CNN}}_{b}$ and ${\mathrm{ANN}}_{b}$, were used as baseline architectures. Changes in the classifier performance for no data standardization, sample-wise standardization, and feature-wise standardization were analyzed using the baseline architectures. The sample- and feature-wise standardization methods involved subtraction of the mean followed by division of the standard deviation calculated either per sample or feature, respectively. Additionally, we modified network parameters, such as kernel size, in the convolutional layer of the CNN and number of hidden units in the hidden layer of ANN to visualize impact on classifier performance. We finally used the best performing neural network in both cases and applied CRF postprocessing. Parameters for the CRF algorithm included the size of the smoothness kernel, $\sigma_{\gamma }$, weight of the smoothness kernel, $w_{2}$, and the number of iterations, n, which were optimized in an *ad hoc* fashion.

## 4 Results

**Fig. 3:**
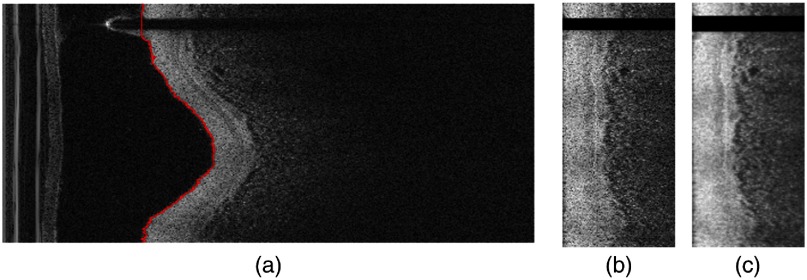
Preprocessing steps on (r,θ) image. (a) Lumen border (red curve) detected using our dynamic programming approach. (b) A-lines were pixel shifted along r to align tissues, with the lumen border on the left-hand side. (c) Pixel-shifted image was filtered with a 2-D Gaussian kernel.

The steps in the preprocessing procedure of a representative IVOCT frame are described in Fig. 3. All images are shown after $\log_{e}$ compression for improved visualization. Pixel shifting successfully aligned the subsurface tissue regions, which improved the subsequent noise reduction filtering, as it reduced filtering across edges of tissue regions.

**Fig. 4:**
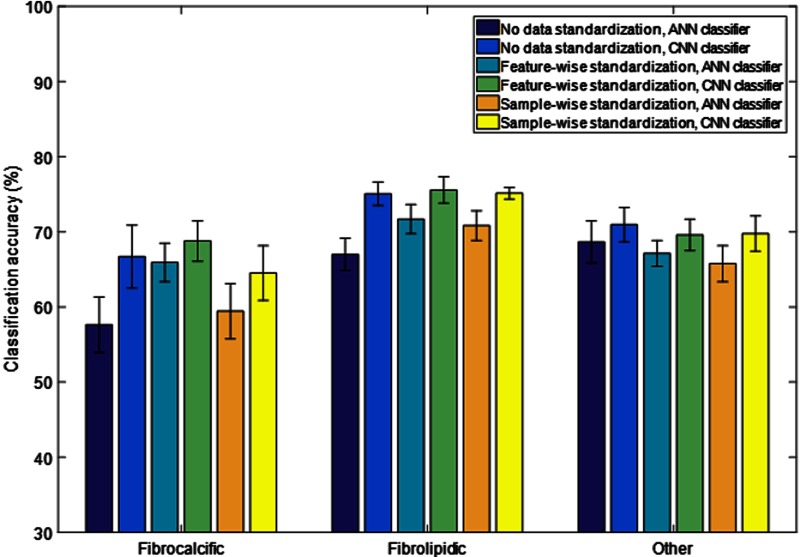
Role of standardization methods on classification accuracy of ${\mathrm{ANN}}_{b}$ and ${\mathrm{CNN}}_{b}$. Results are from 10-fold cross validation without classification noise cleaning. In the case of the ANN, feature-wise standardization gives somewhat better results than other methods. For the CNN, not performing a data standardization step was just as good as performing a feature-wise standardization. See text for details on standardization methods.

**Fig. 5:**
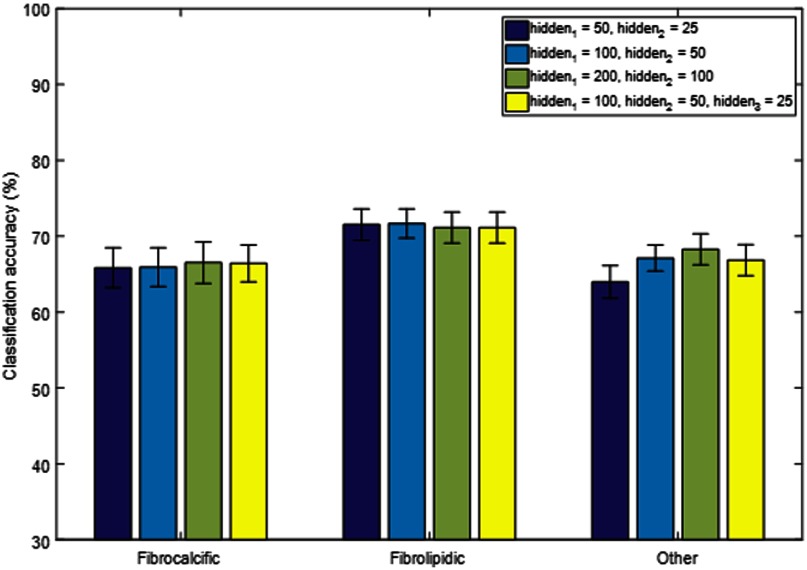
Sensitivity of classification accuracy on ANN design. Starting with the base line architecture (${\mathrm{ANN}}_{b}$: ${\mathrm{hidden}}_{1}=100$, ${\mathrm{hidden}}_{2}=50$), we doubled and halved the number of hidden units and added an additional hidden layer in each new design. The results shown are prior to classification noise cleaning. There is no consistent trend, and ${\mathrm{ANN}}_{b}$ gives good results.

**Fig. 6:**
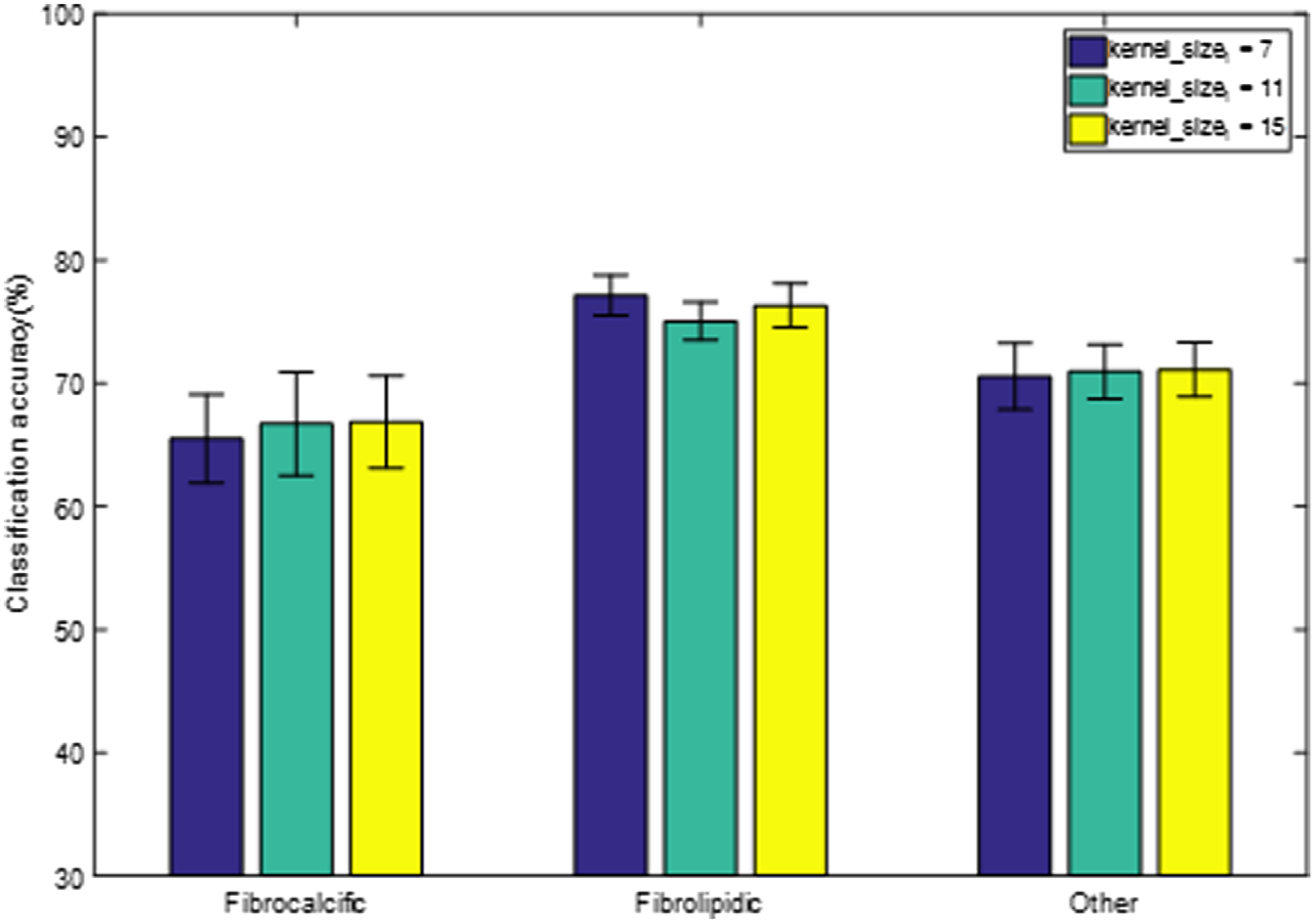
Sensitivity of classification accuracy on CNN design. The baseline kernel size of the first convolutional layer (11 pixels) was increased and decreased to determine the effect on classification accuracy. The results shown are prior to noise cleaning. There is no consistent trend, and ${\mathrm{CNN}}_{b}$ gives good results.

Next, the role of neural network processing parameters on classifier performance was investigated using the baseline classifiers ${\mathrm{ANN}}_{b}$ and ${\mathrm{CNN}}_{b}$, and the following three different data standardization schemes were applied: no data standardization, sample-wise standardization, and feature-wise standardization (Fig. 4). Although there were no large effects, feature-wise standardization worked best for the ANN, whereas eliminating the standardization step was equivalent to feature-wise standardization for CNN. The CNN also tended to have a higher classification accuracy than the ANN for any class and any data standardization method. To determine the potential sensitivity of the network design, experiments were performed with varying network parameters for the ANN and CNN (Figs. 5 and 6, respectively). In the case of the ANN, the number of hidden units in the two hidden layers ${\mathrm{hidden}}_{1}$ and ${\mathrm{hidden}}_{2}$ was modified. We also experimented with the addition of another hidden layer ${\mathrm{hidden}}_{3}$. There was no consistent trend, and the results of ${\mathrm{ANN}}_{b}$ were as good as those of the other configurations. In the case of the CNN, the baseline kernel size of the first convolutional layer of 11 pixels was increased and decreased to determine the effect on classification accuracy. There was no consistent trend, and the results of ${\mathrm{CNN}}_{b}$ were reasonable. In general, changes in network design had relatively little effect on class-wise accuracy. Hence, the baseline networks ${\mathrm{ANN}}_{b}$ and ${\mathrm{CNN}}_{b}$ were used for all subsequent processing.

**Fig. 7:**
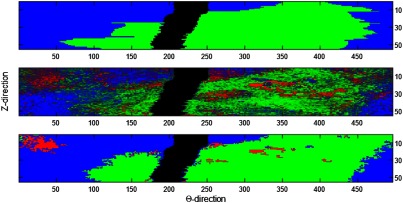
Classification of a lesion with and without classification noise cleaning in *en-face* (θ,z) view. Shown are ground truth labels (top), probability maps for each class from the ${\mathrm{CNN}}_{b}$ classifier (middle), and output of dense CRF processing (bottom). The number of pixels along θ is determined by the number of A-lines collected by the system in one complete rotation of the light probe. The size of the smoothness kernel used was (19, 5) in (θ,z) and the number of iterations n was set to 5. The color code is red (fibrocalcific), green (fibrolipidic), blue (other), and black (guidewire shadow). We used 55 consecutive annotated frames. In this case, ${\mathrm{CNN}}_{b}$ classification accuracy was improved from 65.95% to 90.31% with classification noise cleaning.

It was highly desirable to clean the A-line classification from both networks (Fig. 7). Following noise cleaning, the classification results compared favorably with the annotated labels. We optimized CRF parameters (figure legend) in an *ad hoc* fashion. In general, the CRF results were not very sensitive to parameter optimization. Importantly, despite some remaining errors following noise cleaning, this 7.6-mm vessel segment was clearly dominated by a fibrolipidic lesion, which would be of interest to the clinician. Similar results were obtained using ANN_b_ (not shown).

Confusion matrices for both networks with and without noise cleaning are reported in Tables 2 and 3, respectively. The noise cleaning step improved the class-wise classification accuracy by 10% to 15%. For the fibrocalcific, fibrolipidic, and other classes, the CNN had comparable or better sensitivity and specificity than the ANN (0.80 versus 0.78, 0.85 versus 0.85, and 0.84 versus 0.81, respectively, and 0.95 versus 0.93, 0.92 versus 0.91, and 0.92 versus 0.92, respectively). The types of errors are important. For example, a lipidous lesion should not be misinterpreted as a calcification because it would be undesirable to perform a procedure to modify a calcification (e.g., atherectomy) on a lipidous lesion. As compared with ANN, CNN reduced this type of error by 40%.

**Table 2:** Confusion matrix for A-line classification using the ${\mathrm{ANN}}_{b}$ (top) and ${\mathrm{CNN}}_{b}$ (bottom) prior to noise cleaning. Numbers in brackets indicate the mean and standard error (in percentage) across all folds. Overall classification accuracy is 67.31% and 70.29% with the ANN and CNN approaches, respectively.

	Predicted fibrocalcific	Predicted fibrolipidic	Predicted other
True fibrocalcific	215859 (65.92, 2.57)	57699 (18.38, 1.90)	56167 (15.70, 1.35)
True fibrolipidic	55659 (13.92, 1.47)	306421 (71.68, 1.94)	62685 (14.40, 1.42)
True other	196848 (15.77, 0.96)	229873 (17.11, 1.31)	834594 (67.12, 1.72)
	Predicted fibrocalcific	Predicted fibrolipidic	Predicted other
True fibrocalcific	227402 (66.70, 4.18)	57567 (19.31, 3.13)	44756 (13.99, 1.82)
True fibrolipidic	54504 (11.39, 0.93)	314583 (75.04, 1.55)	55678 (13.56, 1.25)
True other	153874 (11.81, 1.23)	232479 (17.25, 1.69)	874962 (70.94, 2.28)

**Table 3:** Confusion matrix for A-line classification using the ${\mathrm{ANN}}_{b}$ (top) and ${\mathrm{CNN}}_{b}$ (bottom) after noise cleaning. Numbers in brackets indicate the mean and standard error (in percentage) across all folds. The F1 scores for fibrocalcific, fibrolipidic, and other classes were 0.72, 0.77, and 0.86 for the ANN and 0.76, 0.78, and 0.89 for the CNN. Prior to classification noise cleaning, accuracy was 67.31% and 70.29% for the ANN and CNN, respectively.

	Predicted fibrocalcific	Predicted fibrolipidic	Predicted other
True fibrocalcific	259039 (79.37, 2.88)	29870 (9.14, 1.50)	40816 (11.49, 1.91)
True fibrolipidic	20174 (4.99, 1.28)	360646 (83.36, 2.57)	43405 (11.64, 2.27)
True other	110605 (8.39, 1.37)	125106 (9.19, 1.13)	1025604 (82.42, 2.23)
	Predicted fibrocalcific	Predicted fibrolipidic	Predicted other
True fibrocalcific	262548 (77.69, 4.06)	30573 (11.63, 3.80)	36604 (10.67, 2.15)
True fibrolipidic	19740 (2.96, 0.78)	363593 (86.49, 2.31)	41432 (10.55, 2.28)
True other	80148 (5.82, 1.62)	114219 (8.86, 1.21)	1066948 (85.31, 2.50)

Overall, we found that the CNN performs statistically significantly better than the ANN for this task. A two-tailed paired t-test was conducted with the null hypothesis that the means of the error rates of both learning algorithms are equal. A p-value of 0.00027 was obtained, allowing us to reject the null hypothesis. This test was conducted on the classification results prior to noise cleaning by the CRF. We also found that the ANN had higher error rate as compared to the CNN across all folds.

**Fig. 8:**
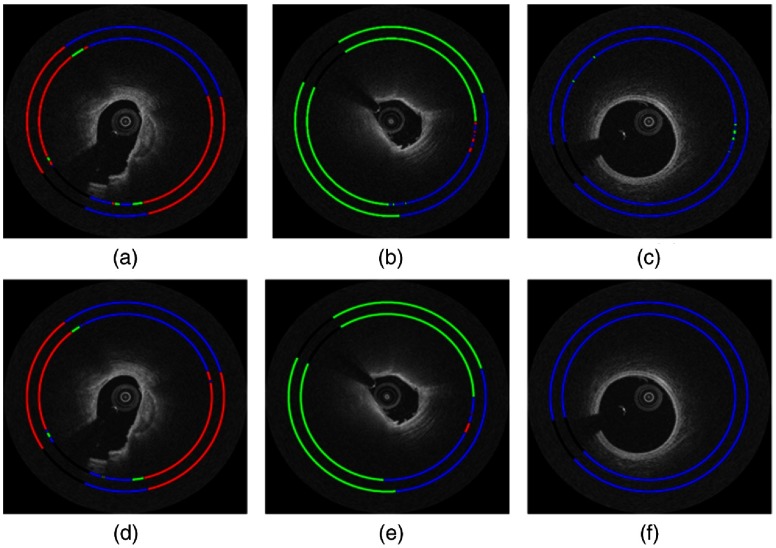
Classification with ${\mathrm{ANN}}_{b}$ [in (a), (b), and (c)] and ${\mathrm{CNN}}_{b}$ [in (d), (e), and (f)] following classification noise cleaning. In this (x,y) view, classification (inner ring) is compared to ground truth labels (outer ring). The color code is red (fibrocalcific), green (fibrolipidic), blue (other), and black (guidewire shadow).

**Fig. 9:**
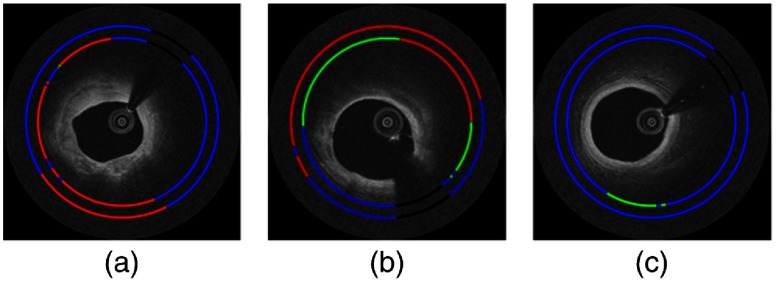
Example frames where the ${\mathrm{CNN}}_{b}$ classifier mispredicts class ownership for a group of A-lines. Experts identified that there are labeling errors in (a), i.e., A-lines from 8 o’clock to 11 o’clock in (a) are indeed fibrocalcific. Additionally, they have identified that A-lines from 9 o’clock to 12 o’clock in (b) are hard to call between fibrocalcific and fibrolipidic classes and could be better labeled as a mixed class. Finally, an example of a true error (classifying other A-lines as fibrolipidic) is shown in (c). In this (x,y) view, classification (inner ring) is compared to ground truth labels (outer ring). The color code is red (fibrocalcific), green (fibrolipidic), blue (other), and black (guidewire shadow).

**Fig. 10:**
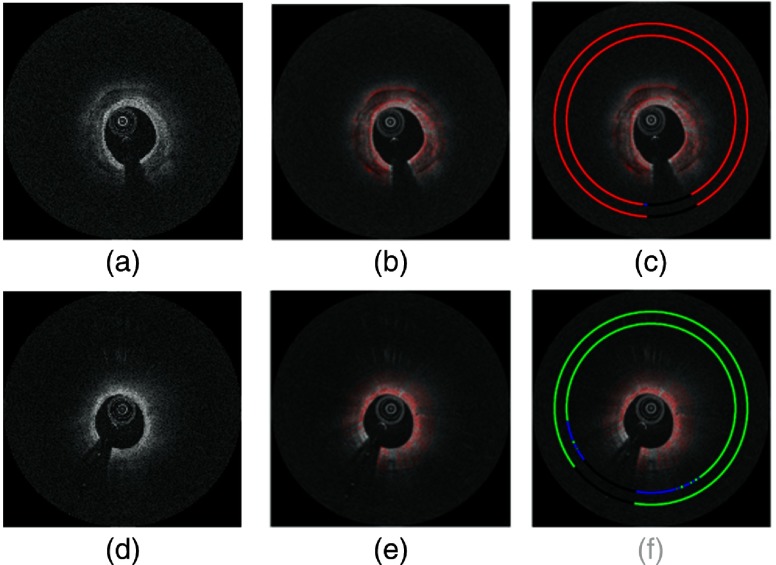
Class saliency maps showing image regions most discriminative for fibrocalcific (a, b, and c) and fibrolipidic (d, e, and f) plaques to the ${\mathrm{CNN}}_{b}$ classifier. Images shown here are raw IVOCT image in (x,y) view (a, d), saliency maps overlaid in red on the IVOCT images (b, e), and final algorithm predictions and ground truth labels overlaid as inner ring and outer ring, respectively, on the image (c, f). Note high saliency at telltale edges of calcification and diffuse edges of lipidous lesion.

As shown by the classification results in Fig. 8, both ANN and CNN perform well, but close examination showed that the CNN agreed more favorably to the annotated labels. The large calcification [Figs. 8(a) and 8(d)] demonstrates the visual characteristics described in the consensus document,^1^ that is, the calcium plaque had sharp front and back edges and appears as a signal-poor region. Similarly, the fibrolipidic region [Figs. 8(b) and 8(e)] had clear characteristics of lipids, that is, diffuse borders with high absorption. The results of the CNN classifier on a few erroneous frames are shown in Fig. 9. Class saliency maps show regions most discriminative for lipid and calcium (Fig. 10). The characteristic edges of the calcification are highlighted in Fig. 10(b), and the diffuse signal decay due to lipids is shown Fig. 10(e).

## 5 Discussion

Although overall A-line classification accuracy is about 82%, the learning methods applied to IVOCT could lead to clinically useful results. During an intervention, a cardiologist is interested in plaque deposits much larger than single A-lines. For example, the interventional cardiologist must identify large circumferential calcifications that might hamper stent deployment so that she can apply an appropriate plaque modification strategy. Alternatively, the presence of a large lipidous plaque requires the use of a longer stent that does not end in a lipidous lesion. Neither of these scenarios require high-resolution accuracy, motivating us to use CRF noise cleaning on the neural network results. Typically, results similar to those in Fig. 7 are obtained where there is no ambiguity in a fibrolipidic segment even though the A-line accuracy of this segment was 87.31%. Processing time suggests that live-time clinical usage is possible. Computation time per frame was 0.3 s (preprocessing), 0.02 s (classification), and 0.002 s (classification noise cleaning). Thus, the total processing time was <1  s using a standard laptop with a GPU (HP Pavilion 15t, NVIDIA GTX 950M). Therefore, the overall computation time for a 500-frame pullback will be completed within a few seconds with the use of a more powerful computer system.

A-lines provide a natural way to analyze IVOCT data that should aid the performance of a learning system. First, the IVOCT system acquires data in a radial fashion, one A-line at a time. Analyzing A-lines avoids the issue of spatially dependent interpolation effects that arise in Cartesian (x,y) images. Second, tissues are naturally ordered with respect to distance from the lumen, for example, a fibrocalcific region consists of a fibrous layer followed by calcification and a normal region consisting of three layers: the intima, media, and adventitia. This also provides motivation for employing an ANN for this task. Third, the order of an A-line captures the reduction of backscattered light due to absorption of light in tissue structures. Fourth, classifying A-lines simplifies the learning task to categorize an A-line into one of three classes, which should reduce the number of required training samples as compared with that of a more complex learning problem, for example, semantic segmentation of all voxels in a pullback. Fifth, A-line classification is useful to identify the extent of a lesion across the pullback volume. It will be interesting to join this classification scheme with traditional image segmentation methods.

A cross-validation procedure over held-out pullbacks was adopted to assess classifier performance. This method is much superior to grouping all images together and leaving out images from the dataset for testing. The latter method gives unnaturally good performance as there can be considerable correlation between images of a single lesion. Additionally, a cross-validation procedure gives a better approximation of generalization error than with a single held-out test dataset.

Few motivations exist for comparing an ANN and CNN at this task. First, since our method classified one A-line at a time, the number of input pixels was reasonable to use a fully connected ANN. Second, and as mentioned earlier, tissues have a natural order along the depth of an A-line. We anticipated that an ANN would capture this order by considering individual pixel values as features. Third, since the thickness of the fibrous layer in plaques was variable within the dataset, we hypothesized that a CNN would perform well, due to the spatial invariance property of the convolution operator.

There were some interesting observations regarding the learning system structure, processing, and types of errors. Changes in the design of the CNN or ANN did not largely impact the performance (Figs. 5 and 6), indicating that the initial architectures were relatively stable on this dataset. Because the dataset was obtained from the same site and IVOCT system, the standardization step was unnecessary. However, when working with datasets from different sites and systems, it might be useful to standardize the datasets separately. In regard to the type of errors made by the classifier, it is clinically desirable to have fewer false positive calls for the fibrocalcific class. The reason is that the treatment strategy for fibrocalcific plaques is to use an atherectomy device that grinds through plaque, potentially damaging fibrolipidic and other regions. We found that the CNN made fewer such false positive calls as compared to the ANN.

Saliency maps for calcium and lipid plaques show that the network learns features that are consistent with those described in the consensus document^1^ for IVOCT image interpretation. Qualitatively, a calcium plaque has a signal-poor region with sharp front and/or back edges, whereas lipid plaques have highly attenuating regions with diffuse borders. The saliency map for fibrocalcific plaque shows that the pixels belonging to the front and back edges of a calcium plaque were most responsible for calling the A-line as fibrocalcific. Similarly, the pixels belonging to the blurred edge of the lipid plaque along with pixels far away from the plaque boundary contribute the most to calling an A-line as fibrolipidic. Although IVOCT can delineate regions of the vessel wall into several other categories, such as macrophage accumulation, intimal vasculature, and thrombus, this analysis was restricted to the above-mentioned class types. However, it is possible to extend this methodology to class types other than those mentioned in this paper.

Labeling is time-consuming, and some tissues are difficult to call, likely leading to noisy A-line labeling, which would degrade performance metrics and potentially degrade the learned model. With this in mind, we are greatly encouraged by these results. Interestingly, we identified cases (e.g., Fig. 9) where the CNN results could lead experts to change their original labels, suggesting the possibility of active learning with a second pass of the dataset to possibly modify the labels.
